# Dimethyl 2,2′-[(4-oxo-2-phenyl-4*H*-chromene-5,7-diyl)dioxy]diacetate: a more densely packed polymorph

**DOI:** 10.1107/S1600536809001020

**Published:** 2009-01-14

**Authors:** Angannan Nallasivam, Munirathinam Nethaji, Nagarajan Vembu, Buckle Jaswant, Nagarajan Sulochana

**Affiliations:** a Department of Chemistry, National Institute of Technology, Tiruchirappalli 620 015, India; b Department of Inorganic and Physical Chemistry, Indian Institute of Science, Bangalore 560 012, India; c Department of Chemistry, Urumu Dhanalakshmi College, Tiruchirappalli 620 019, India; d Department of Chemistry, Government Arts College, Karur 639 005, India

## Abstract

The title mol­ecule, C_21_H_18_O_8_, crystallizes in two crystal polymorphs, see also Nallasivam, Nethaji, Vembu & Jaswant [*Acta Cryst.* (2009), E**65**, o314–o315]. The mol­ecules of both polymorphs differ by the conformation of the oxomethyl­acetate groups. The title mol­ecules are rather planar compared to the mol­ecules of the other polymorph. In the title mol­ecule, one of the oxomethyl­acetate groups is disordered (occupancies of 0.6058/0.3942). The structures of both polymorphs are stabilized by C—H⋯O and C—H⋯π inter­actions. Due to the planarity of the title mol­ecules and similar inter­molecular inter­actions, the title mol­ecules are more densely packed than those of the other polymorph.

## Related literature

For a more detailed description of the two polymorphs, see: Nallasivam *et al.* (2009 ▶). For related structures, see: Wang, Fang *et al.* (2003 ▶); Wang, Zheng *et al.* (2003 ▶). For hydrogen bonding, see: Desiraju & Steiner (1999 ▶).
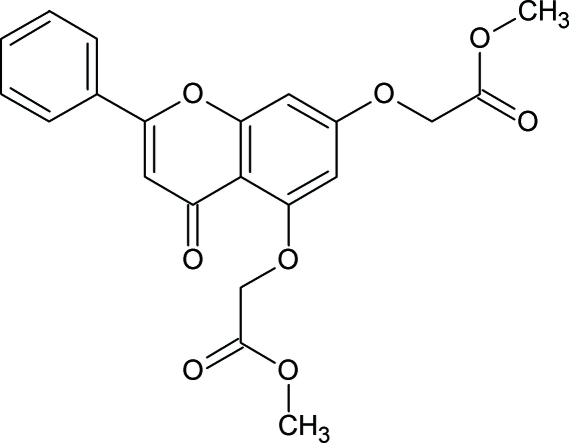


## Experimental

### 

#### Crystal data

C_21_H_18_O_8_
*M* *_r_* = 398.35
Triclinic, 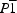

*a* = 7.4290 (15) Å
*b* = 9.2582 (19) Å
*c* = 13.480 (3) Å
α = 84.232 (3)°
β = 88.775 (4)°
γ = 82.982 (3)°
*V* = 915.5 (3) Å^3^
*Z* = 2
Mo *K*α radiation
μ = 0.11 mm^−1^
*T* = 293 (2) K
0.34 × 0.28 × 0.22 mm

#### Data collection

Bruker SMART APEX CCD diffractometer
Absorption correction: multi-scan (*SADABS*; Sheldrick, 1998 ▶) *T*_min_ = 0.963, *T*_max_ = 0.975
9755 measured reflections
3781 independent reflections
2887 reflections with *I* > 3σ(*I*)
*R*_int_ = 0.016

#### Refinement

*R*[*F* ^2^ > 2σ(*F* ^2^)] = 0.059
*wR*(*F* ^2^) = 0.118
*S* = 2.47
3781 reflections
268 parameters
H-atom parameters constrained
Δρ_max_ = 0.40 e Å^−3^
Δρ_min_ = −0.26 e Å^−3^

### 

Data collection: *SMART* (Bruker, 1999 ▶); cell refinement: *SAINT* (Bruker, 1999 ▶); data reduction: *SAINT*; program(s) used to solve structure: *SHELXS97* (Sheldrick, 2008 ▶); program(s) used to refine structure: *JANA2000* (Petříček *et al.*, 2000 ▶); molecular graphics: *PLATON* (Spek, 2003 ▶); software used to prepare material for publication: *SHELXL97* (Sheldrick, 2008 ▶) and *JANA2000*.

## Supplementary Material

Crystal structure: contains datablocks I, global. DOI: 10.1107/S1600536809001020/fb2129sup1.cif

Structure factors: contains datablocks I. DOI: 10.1107/S1600536809001020/fb2129Isup2.hkl

Additional supplementary materials:  crystallographic information; 3D view; checkCIF report

## Figures and Tables

**Table 1:** Hydrogen-bond geometry (Å, °)

*D*—H⋯*A*	*D*—H	H⋯*A*	*D*⋯*A*	*D*—H⋯*A*
C8—H8⋯O27^i^	0.93	2.38	3.304 (2)	169
C12—H12⋯O1	0.93	2.33	2.664 (2)	101
C15—H15⋯O21B^ii^	0.93	2.46	3.31 (6)	153
C25—H25B⋯O17^iii^	0.97	2.56	3.447 (3)	153
C29—H29B⋯O17^iv^	0.96	2.50	3.403 (3)	156
C29—H29C⋯O21B^iv^	0.96	2.55	3.26 (6)	131
C23B—H23BB⋯*Cg*1^v^	0.96	2.76	3.67 (6)	159 (6)

Symmetry codes: (i) 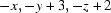; (ii) 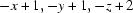; (iii) 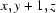; (iv) 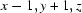; (v) 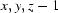. *Cg*1 is the centroid of the C11–C16 phenyl ring.

**Fig. 1.**
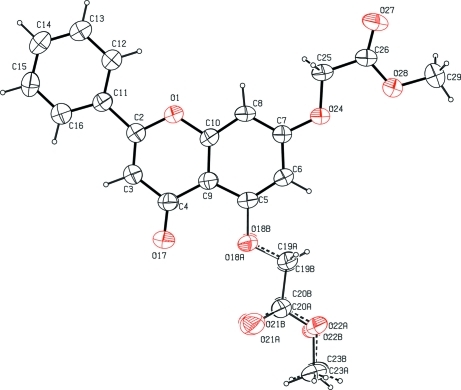
The asymmetric unit of the title compound with the atoms labelled and displacement ellipsoids depicted at the 50% probability level for all non-H atoms. H-atoms are drawn as spheres of arbitrary radius.

## supplementary crystallographic information

### Comment

The importance of the benzopyrans and their derivatives is described in Nallasivam *et al.* (2009).

The chromene ring is almost planar and similar to that found in the related chromene derivatives (Wang, Zheng *et al.*, 2003; Wang, Fang *et al.*, 2003). The total puckering amplitude of the chromene ring is 0.040 (2)Å. The interplanar angle between the chromene ring and the 2-phenyl ring is 2.90 (6)° thereby indicating the almost coplanar arrangement (Fig. 1). The oxomethylacetate substituent at C7 is slightly distorted from coplanarity as discerned from the interplanar angle of 12.7 (1)°. Such a calculation for the oxomethylacetate group at C5 is not done due to disorder.

The crystal structure is stabilized by the interplay of C–H···O, C–H···π interactions (Tab. 1) as well as π···π-electron interactions. The H-bond distances agree with those reported in literature (Desiraju & Steiner, 1999). There is a π···π-electron interaction between the rings C5\C6···\C10 *Cg*2  and C11\C12···\C16 [1-*x*, 2-*y*, 2-*z*] whose centroids are at the distance 3.714 (1) Å.

### Experimental

Into the round bottom flask a suspension of chrysin (3.93 mmol, 1 g) and potassium carbonate (11.81 mmol, 1.64 g) were deposited and to this mixture dimethylformamide (10 ml) was added. The reaction mixture was heated to 383 K and maintained at this temperature for 2–3 hrs. The reaction mixture was cooled to 353 K. Methyl chloroacetate (15.74 mmol, 1.70 g) was slowly added to the reaction mixture with the help of a dropping funnel. The reaction mixture was kept for 8–9 hrs at 353 K while the reaction was monitored by high pressure liquid chromatography. Once the reaction was completed, the reaction mixture was quenched with water and stirred for 30–45 min at 303 K. The obtained solid was filtered and washed with plenty of water followed by methanol. The wet cake was dried under vacuum at 343 K. The crude product of the title compound, *i. e.* the more densely packed polymorph, was dissolved in dichloromethane (10 ml) and mixed with equal amount of n-hexane. The clear solution was kept aside for a week without stirring. Diffraction quality prism shaped crystals with average size about 0.30 mm along the longest edge were obtained. The crystals were filtered and washed with n-hexane and dried under vacuum at 70°C. Yield: 85%

### Refinement

Though the hydrogen atoms were observable in the difference electron density maps they were situated into the idealized positions and refined in the riding mode approximation. The following constraints have been applied: C–H = 0.93, 0.97 and 0.96Å for aryl, methylene and methyl H, respectively. *U*_iso_(H)=1.2U_eq_(C) for the aryl and methylene H and *U*_iso_(H)=1.5U_eq_(C) for the methyl H. A considerably elongated displacement parameter of the atom O21 and electron density maxima in the vicinity of the disordered chain atoms indicated disorder. This disorder has been modelled by two fragments whose geometry was assumed to be equal with relatively same displacement parameters that differed only by their orientation that was refined. At the beginning, the atoms of the disordered fragment were refined isotropically while their occupational parameters were refined. The occupational parameters converged to the values 0.394 (4) and 0.606 (4), respectively. In the next stage, the occupational parameters were fixed while the non-hydrogen atoms of the disordered atoms were refined anisotropically. The plausibility of the result follows from the planarity of the disordered fragments C19A\C20A\O21A\O22A and C19B\C20B\O21B\O22B with  maximal deviations from planarity that equal to 0.006 (7) Å for C20A and 0.007 (65)Å for C20B.

### Crystal data

**Table:**

C_21_H_18_O_8_	*Z* = 2
*M**_r_* = 398.35	*F*(000) = 416
Triclinic, *P*1	*D*_x_ = 1.445 Mg m^−^^3^
Hall symbol: -P 1	Melting point = 411–414 K
*a* = 7.4290 (15) Å	Mo *K*α radiation, λ = 0.71073 Å
*b* = 9.2582 (19) Å	Cell parameters from 574 reflections
*c* = 13.480 (3) Å	θ = 1.5–26.5°
α = 84.232 (3)°	µ = 0.11 mm^−^^1^
β = 88.775 (4)°	*T* = 293 K
γ = 82.982 (3)°	Rectangular, colourless
*V* = 915.5 (3) Å^3^	0.34 × 0.28 × 0.22 mm

### Data collection

**Table:**

Bruker SMART APEX CCD diffractometer	3781 independent reflections
Radiation source: fine-focus sealed tube	2887 reflections with *I* > 3σ(*I*)
graphite	*R*_int_ = 0.016
Detector resolution: 0.3 pixels mm^-1^	θ_max_ = 27.4°, θ_min_ = 1.5°
ω scans	*h* = −9→9
Absorption correction: multi-scan (*SADABS*; Sheldrick, 1998)	*k* = −11→11
*T*_min_ = 0.963, *T*_max_ = 0.975	*l* = −17→17
9755 measured reflections	

### Refinement

**Table:**

Refinement on *F*^2^	Primary atom site location: structure-invariant direct methods
Least-squares matrix: full	Secondary atom site location: difference Fourier map
*R*[*F*^2^ > 2σ(*F*^2^)] = 0.059	Hydrogen site location: difference Fourier map
*wR*(*F*^2^) = 0.118	H-atom parameters constrained
*S* = 2.47	Weighting scheme based on measured s.u.'s *w* = 1/[σ^2^(*I*) + 0.0004*I*^2^]
3781 reflections	(Δ/σ)_max_ = 0.018
268 parameters	Δρ_max_ = 0.40 e Å^−^^3^
0 restraints	Δρ_min_ = −0.26 e Å^−^^3^
73 constraints	

### Fractional atomic coordinates and isotropic or equivalent isotropic displacement parameters (Å^2^)

**Table:**

	*x*	*y*	*z*	*U*_iso_*/*U*_eq_	Occ. (<1)
O1	0.30663 (16)	0.96130 (13)	1.06056 (8)	0.0466 (4)	
C2	0.3857 (2)	0.82585 (19)	1.09503 (13)	0.0431 (6)	
C3	0.4037 (2)	0.7161 (2)	1.03579 (13)	0.0481 (7)	
H3	0.462382	0.628089	1.064955	0.0577*	
C4	0.3379 (2)	0.73194 (19)	0.93493 (13)	0.0447 (6)	
C5	0.1751 (2)	0.92338 (19)	0.80498 (13)	0.0440 (6)	
C6	0.0924 (2)	1.06310 (19)	0.78160 (13)	0.0485 (7)	
H6	0.040296	1.087598	0.71911	0.0582*	
C7	0.0866 (2)	1.16664 (19)	0.85047 (13)	0.0431 (6)	
C8	0.1604 (2)	1.13114 (18)	0.94310 (12)	0.0425 (6)	
H8	0.156976	1.200597	0.988599	0.051*	
C9	0.2523 (2)	0.87883 (19)	0.90045 (12)	0.0412 (6)	
C10	0.2396 (2)	0.98822 (19)	0.96535 (12)	0.0404 (6)	
C11	0.4412 (2)	0.82212 (19)	1.19964 (13)	0.0442 (6)	
C12	0.4040 (3)	0.9455 (2)	1.25078 (13)	0.0566 (7)	
H12	0.343711	1.031075	1.2191	0.0679*	
C13	0.4559 (3)	0.9425 (2)	1.34867 (14)	0.0662 (9)	
H13	0.430455	1.025051	1.382886	0.0795*	
C14	0.5450 (3)	0.8187 (2)	1.39655 (15)	0.0682 (9)	
H14	0.579756	0.818894	1.462394	0.0818*	
C15	0.5827 (3)	0.6948 (2)	1.34736 (14)	0.0639 (8)	
H15	0.64295	0.610021	1.380221	0.0767*	
C16	0.5311 (3)	0.6965 (2)	1.24944 (14)	0.0540 (7)	
H16	0.557134	0.612394	1.216813	0.0648*	
O17	0.3521 (2)	0.62674 (13)	0.88447 (9)	0.0631 (5)	
O24	0.00341 (17)	1.30050 (13)	0.81551 (9)	0.0543 (5)	
C25	0.0005 (3)	1.41742 (19)	0.87637 (13)	0.0510 (7)	
H25a	−0.042676	1.386777	0.942667	0.0612*	
H25b	0.122418	1.44296	0.881842	0.0612*	
C26	−0.1214 (3)	1.5488 (2)	0.83259 (14)	0.0515 (7)	
O27	−0.1465 (2)	1.65854 (16)	0.87223 (11)	0.0857 (7)	
O28	−0.19680 (19)	1.52877 (14)	0.74809 (10)	0.0629 (5)	
C29	−0.3222 (3)	1.6483 (2)	0.70402 (17)	0.0749 (9)	
H29a	−0.258565	1.731194	0.685106	0.1124*	
H29b	−0.415753	1.673972	0.751552	0.1124*	
H29c	−0.375575	1.619165	0.646084	0.1124*	
O18a	0.1645 (15)	0.8187 (11)	0.7443 (6)	0.0454 (13)	0.3942
C19a	0.1208 (13)	0.8711 (7)	0.6431 (7)	0.0539 (9)	0.3942
H19aa	0.208107	0.93525	0.616762	0.0647*	0.3942
H19ba	−0.002303	0.920137	0.640124	0.0647*	0.3942
C20a	0.1342 (9)	0.7395 (6)	0.5854 (5)	0.0499 (10)	0.3942
O21a	0.2023 (7)	0.6206 (5)	0.6126 (4)	0.0810 (12)	0.3942
O22a	0.0619 (16)	0.7758 (10)	0.4972 (6)	0.0728 (8)	0.3942
C23a	0.0795 (19)	0.6627 (13)	0.4291 (6)	0.0683 (15)	0.3942
H23aa	−0.019173	0.680175	0.38296	0.1025*	0.3942
H23ba	0.192314	0.664225	0.393153	0.1025*	0.3942
H23ca	0.077136	0.568838	0.466291	0.1025*	0.3942
O18b	0.209 (9)	0.823 (7)	0.734 (4)	0.0454 (12)	0.6058
C19b	0.108 (9)	0.860 (7)	0.643 (4)	0.0539 (9)	0.6058
H19ab	0.136	0.955	0.613	0.0647*	0.6058
H19bb	−0.020	0.861	0.658	0.0647*	0.6058
C20b	0.169 (9)	0.745 (7)	0.575 (4)	0.0499 (10)	0.6058
O21b	0.298 (9)	0.657 (7)	0.585 (4)	0.0810 (13)	0.6058
O22b	0.058 (9)	0.756 (7)	0.500 (4)	0.0728 (9)	0.6058
C23b	0.109 (9)	0.660 (7)	0.422 (4)	0.0683 (14)	0.6058
H23ab	0.002	0.641	0.390	0.1025*	0.6058
H23bb	0.188	0.705	0.375	0.1025*	0.6058
H23cb	0.171	0.569	0.452	0.1025*	0.6058

### Atomic displacement parameters (Å^2^)

**Table:**

	*U*^11^	*U*^22^	*U*^33^	*U*^12^	*U*^13^	*U*^23^
O1	0.0634 (8)	0.0405 (7)	0.0362 (7)	−0.0007 (6)	−0.0085 (6)	−0.0092 (5)
C2	0.0488 (11)	0.0395 (10)	0.0406 (10)	−0.0030 (8)	−0.0026 (8)	−0.0041 (8)
C3	0.0613 (12)	0.0393 (11)	0.0422 (11)	0.0007 (9)	−0.0052 (9)	−0.0034 (9)
C4	0.0550 (12)	0.0397 (10)	0.0407 (10)	−0.0067 (9)	−0.0008 (9)	−0.0092 (8)
C5	0.0542 (11)	0.0415 (11)	0.0382 (10)	−0.0048 (9)	−0.0028 (9)	−0.0141 (8)
C6	0.0610 (12)	0.0460 (11)	0.0389 (10)	0.0007 (9)	−0.0115 (9)	−0.0126 (9)
C7	0.0493 (11)	0.0387 (10)	0.0415 (11)	0.0004 (8)	−0.0071 (8)	−0.0101 (8)
C8	0.0508 (11)	0.0382 (10)	0.0405 (10)	−0.0035 (8)	−0.0024 (9)	−0.0150 (8)
C9	0.0488 (11)	0.0389 (10)	0.0369 (10)	−0.0053 (8)	−0.0006 (8)	−0.0084 (8)
C10	0.0463 (11)	0.0428 (10)	0.0330 (10)	−0.0058 (8)	−0.0043 (8)	−0.0070 (8)
C11	0.0501 (11)	0.0446 (11)	0.0387 (10)	−0.0061 (9)	−0.0041 (8)	−0.0063 (8)
C12	0.0733 (14)	0.0501 (12)	0.0450 (12)	0.0026 (10)	−0.0118 (10)	−0.0074 (9)
C13	0.0943 (17)	0.0586 (14)	0.0456 (12)	0.0007 (12)	−0.0151 (12)	−0.0133 (10)
C14	0.0906 (16)	0.0720 (15)	0.0415 (12)	−0.0047 (13)	−0.0191 (11)	−0.0061 (11)
C15	0.0808 (15)	0.0557 (13)	0.0513 (13)	0.0012 (11)	−0.0194 (11)	0.0061 (11)
C16	0.0660 (13)	0.0469 (12)	0.0483 (12)	−0.0028 (10)	−0.0051 (10)	−0.0050 (9)
O17	0.0964 (11)	0.0410 (8)	0.0521 (8)	0.0037 (7)	−0.0148 (8)	−0.0163 (7)
O24	0.0758 (9)	0.0410 (7)	0.0454 (8)	0.0097 (7)	−0.0183 (7)	−0.0176 (6)
C25	0.0640 (13)	0.0451 (11)	0.0462 (11)	−0.0016 (9)	−0.0113 (9)	−0.0188 (9)
C26	0.0646 (13)	0.0428 (11)	0.0482 (11)	−0.0004 (9)	−0.0069 (10)	−0.0169 (9)
O27	0.1173 (13)	0.0557 (9)	0.0844 (11)	0.0210 (9)	−0.0296 (10)	−0.0403 (9)
O28	0.0860 (10)	0.0460 (8)	0.0543 (8)	0.0130 (7)	−0.0227 (7)	−0.0150 (7)
C29	0.0941 (18)	0.0538 (13)	0.0706 (15)	0.0179 (12)	−0.0208 (13)	−0.0041 (11)
O18a	0.064 (4)	0.0384 (9)	0.0340 (10)	−0.0010 (16)	−0.0036 (17)	−0.0123 (6)
C19a	0.0779 (19)	0.0424 (12)	0.0428 (11)	−0.0047 (12)	−0.0164 (12)	−0.0109 (10)
C20a	0.067 (2)	0.0432 (13)	0.0399 (13)	−0.0034 (13)	−0.0088 (14)	−0.0072 (10)
O21a	0.126 (3)	0.0534 (12)	0.0584 (17)	0.025 (2)	−0.026 (2)	−0.0159 (11)
O22a	0.1281 (17)	0.0475 (14)	0.0427 (8)	0.0023 (14)	−0.0274 (10)	−0.0139 (8)
C23a	0.104 (4)	0.0602 (17)	0.0447 (13)	−0.0086 (18)	−0.0085 (16)	−0.0243 (12)
O18b	0.054 (3)	0.0478 (18)	0.0344 (12)	0.0054 (17)	−0.0039 (15)	−0.0159 (11)
C19b	0.0620 (16)	0.0513 (16)	0.0491 (12)	0.0063 (11)	−0.0157 (11)	−0.0211 (11)
C20b	0.0555 (19)	0.0502 (17)	0.0436 (14)	0.0037 (13)	−0.0099 (13)	−0.0131 (12)
O21b	0.074 (3)	0.097 (2)	0.0673 (19)	0.0350 (17)	−0.0209 (19)	−0.0378 (15)
O22b	0.0888 (16)	0.0733 (18)	0.0544 (9)	0.0260 (12)	−0.0302 (9)	−0.0340 (9)
C23b	0.091 (3)	0.070 (2)	0.0462 (15)	0.0046 (19)	−0.0098 (15)	−0.0298 (14)

### Geometric parameters (Å, °)

**Table:**

O1—C2	1.359 (2)	C25—H25a	0.9700
O1—C10	1.375 (2)	C25—H25b	0.970
C2—C3	1.347 (3)	C25—C26	1.502 (2)
C2—C11	1.474 (2)	C26—O27	1.188 (3)
C3—H3	0.9300	C26—O28	1.319 (2)
C3—C4	1.442 (2)	O28—C29	1.444 (2)
C4—C9	1.464 (2)	C29—H29a	0.960
C4—O17	1.236 (2)	C29—H29b	0.960
C5—C6	1.372 (2)	C29—H29c	0.960
C5—C9	1.422 (2)	O18a—C19a	1.431 (13)
C5—O18a	1.340 (10)	C19a—H19aa	0.970
C5—O18b	1.40 (6)	C19a—H19ba	0.970
C6—H6	0.9300	C19a—C20a	1.503 (10)
C6—C7	1.396 (3)	C19a—H19ab	0.86
C7—C8	1.368 (2)	H19ba—C19b	0.93
C7—O24	1.3593 (19)	C20a—O21a	1.181 (7)
C8—H8	0.9300	C20a—O22a	1.310 (11)
C8—C10	1.387 (2)	O22a—C23a	1.453 (15)
C9—C10	1.397 (3)	C23a—H23aa	0.960
C11—C12	1.389 (3)	C23a—H23ba	0.960
C11—C16	1.387 (2)	C23a—H23ca	0.960
C12—H12	0.9300	O18b—C19b	1.43 (8)
C12—C13	1.379 (3)	C19b—H19ab	0.97
C13—H13	0.930	C19b—H19bb	0.97
C13—C14	1.360 (3)	C19b—C20b	1.50 (9)
C14—H14	0.930	C20b—O21b	1.18 (9)
C14—C15	1.379 (3)	C20b—O22b	1.31 (8)
C15—H15	0.930	O22b—C23b	1.45 (9)
C15—C16	1.380 (3)	C23b—H23ab	0.96
C16—H16	0.930	C23b—H23bb	0.96
O24—C25	1.420 (2)	C23b—H23cb	0.96
			
C2—O1—C10	120.73 (14)	H25a—C25—H25b	108.35
O1—C2—C3	120.63 (15)	H25a—C25—C26	109.47
O1—C2—C11	110.99 (15)	H25b—C25—C26	109.47
C3—C2—C11	128.38 (15)	C25—C26—O27	122.01 (18)
C2—C3—H3	114.72	C25—C26—O28	113.56 (16)
C2—C3—C4	123.30 (15)	O27—C26—O28	124.42 (17)
H3—C3—C4	121.98	C26—O28—C29	116.61 (15)
C3—C4—C9	114.63 (16)	O28—C29—H29a	109.5
C3—C4—O17	120.99 (15)	O28—C29—H29b	109.47
C9—C4—O17	124.36 (16)	O28—C29—H29c	109.47
C6—C5—C9	121.19 (17)	H29a—C29—H29b	109.5
C6—C5—O18a	121.2 (4)	H29a—C29—H29c	109.5
C6—C5—O18b	122 (2)	H29b—C29—H29c	109.5
C9—C5—O18a	117.0 (4)	C5—O18a—C19a	114.8 (7)
C9—C5—O18b	116 (2)	C5—O18a—C19b	119 (3)
C5—C6—H6	118.85	O18a—C19a—H19aa	109.5
C5—C6—C7	120.54 (16)	O18a—C19a—H19ba	109.5
H6—C6—C7	120.61	O18a—C19a—C20a	106.9 (6)
C6—C7—C8	121.07 (15)	O18a—C19a—C20b	109 (2)
C6—C7—O24	113.49 (14)	H19aa—C19a—H19ba	112.0 (6)
C8—C7—O24	125.43 (16)	H19aa—C19a—C20a	109.5 (8)
C7—C8—H8	120.81	H19ba—C19a—C20a	109.5 (8)
C7—C8—C10	117.06 (16)	C19a—C20a—O21a	126.3 (7)
H8—C8—C10	122.13	C19a—C20a—O22a	110.0 (6)
C4—C9—C5	126.02 (16)	O21a—C20a—O22a	123.7 (7)
C4—C9—C10	119.19 (15)	C20a—O22a—C23a	116.3 (8)
C5—C9—C10	114.79 (15)	O22a—C23a—H23aa	109.5
O1—C10—C8	113.17 (15)	O22a—C23a—H23ba	109.5
O1—C10—C9	121.50 (14)	O22a—C23a—H23ca	109.5
C8—C10—C9	125.33 (15)	H23aa—C23a—H23ba	109.5
C2—C11—C12	120.37 (15)	H23aa—C23a—H23ca	109.5
C2—C11—C16	121.24 (16)	H23ba—C23a—H23ca	109.5
C12—C11—C16	118.39 (16)	C5—O18b—C19a	113 (4)
C11—C12—H12	120.07	C5—O18b—C19b	115 (5)
C11—C12—C13	120.55 (17)	C5—O18b—C19b	115 (5)
H12—C12—C13	119.4	O18b—C19b—H19ab	109
C12—C13—H13	120.44	O18b—C19b—H19bb	109
C12—C13—C14	120.5 (2)	O18b—C19b—C20b	107
H13—C13—C14	119.07	H19ab—C19b—H19bb	112
C13—C14—H14	119.3	H19ab—C19b—C20b	109
C13—C14—C15	119.95 (19)	H19bb—C19b—C20b	109
H14—C14—C15	120.7	C19b—C20b—O21b	126 (6)
C14—C15—H15	119.9	C19b—C20b—O22b	110 (6)
C14—C15—C16	120.03 (18)	O21b—C20b—O22b	124 (6)
H15—C15—C16	120.0	C20b—O22b—C23b	116 (6)
C11—C16—C15	120.57 (18)	O22b—C23b—H23ab	109
C11—C16—H16	119.94	O22b—C23b—H23bb	109
C15—C16—H16	119.49	O22b—C23b—H23cb	109
C7—O24—C25	118.58 (13)	H23ab—C23b—H23bb	109
O24—C25—H25a	109.47	H23ab—C23b—H23cb	109
O24—C25—H25b	109.47	H23bb—C23b—H23cb	109
O24—C25—C26	110.57 (15)		

### Hydrogen-bond geometry (Å, °)

**Table:**

*D*—H···*A*	*D*—H	H···*A*	*D*···*A*	*D*—H···*A*
C8—H8···O27^i^	0.93	2.38	3.304 (2)	169
C12—H12···O1	0.93	2.33	2.664 (2)	101
C15—H15···O21B^ii^	0.93	2.46	3.31 (6)	153
C25—H25B···O17^iii^	0.97	2.56	3.447 (3)	153
C29—H29B···O17^iv^	0.96	2.50	3.403 (3)	156
C29—H29C···O21B^iv^	0.96	2.55	3.26 (6)	131
C23B—H23BB···Cg1^v^	0.96	2.76	3.67 (6)	159 (6)

Symmetry codes: (i) −*x*, −*y*+3, −*z*+2; (ii) −*x*+1, −*y*+1, −*z*+2; (iii) *x*, *y*+1, *z*; (iv) *x*−1, *y*+1, *z*; (v) *x*, *y*, *z*−1.
